# Vaccine alliance building blocks: a conjoint experiment on popular support for international COVID-19 cooperation formats

**DOI:** 10.1007/s11077-021-09435-1

**Published:** 2021-08-11

**Authors:** Pieter Vanhuysse, Michael Jankowski, Markus Tepe

**Affiliations:** 1 Danish Centre for Welfare Studies, Department of Political Science and Public Management, and Danish Institute for Advanced Study, University of Southern Denmark, Campusvej, 55, 5230 Odense, Denmark; 2 Institute for Social Sciences, University of Oldenburg, Ammerländer Heerstraße 114-118, 26129 Oldenburg, Germany

**Keywords:** Vaccine alliance, Vaccine nationalism, Need-based fairness, National self-interest, International cooperation, Conjoint experiment

## Abstract

The design principles of institutions that visibly and significantly affect citizens’ lives are likely to be politically salient. Popular support for these principles is in turn crucial for institutional viability and effectiveness. Transboundary pandemics are a case in point. Understanding citizens’ preferences regarding the design of international alliances set up to mass-produce and distribute vaccines is likely to determine citizens’ subsequent cooperation with vaccination campaigns. This study explores Germans’ preferences for international COVID-19 vaccine alliance design principles. We conducted a conjoint experiment at a recurring cognitive moment in many pandemics’ cycles, between the initial outbreak and a more devastating but still-unknown second wave, when infection rates were very low, yet no policy solutions had been developed. We analyzed preferences regarding four building blocks: (1) alliance composition (size; EU-centrism), (2) alliance distribution rules (joining cost; vaccine allocation), (3) vaccine nationalism (cost per German household; coverage in Germany) and (4) vaccine producer confidence (origin; type). Distribution rules, political ideology and personal perceptions of pandemic threat matter little. But a larger alliance size and dominant EU-country composition increase alliance support. And vaccine nationalism is key: support increases with both lower costs and larger coverage for own-nation citizens. Moreover, support goes down for Chinese and American producers and increases for Swiss and especially own-nation producers. In sum, a realist and technocratic outlook is warranted at the cognitive stage in pandemic cycles when no solutions have been found, yet the worst already seems to be over, as national self-interest reigns supreme in popular attitudes.

**Supplementary Information:**

The online version contains supplementary material available at 10.1007/s11077-021-09435-1.

## Introduction

A large literature in political economy and public policy shows that public opinion is an important determinant of policies and institutions (Burstein, 2014; Wlezien & Soroka, 2016). Public support for the specific design principles of institutions that clearly and significantly affect citizens’ lives is likely to be not just especially politically salient, but also crucial for the subsequent viability and effectiveness of these institutions. International cooperation efforts to produce and distribute potentially life-saving vaccines during global pandemics provide one key illustration of the political importance of institutional design specifics. Pandemics threaten citizens’ health in tangible ways, leading them to pay particular attention to how national politicians build international alliances to produce and distribute vaccines that may safeguard public health. Moreover, popular preferences regarding the specific building blocks of such alliances are likely to be crucial for citizens’ acceptance of, and cooperation with, subsequent vaccination campaigns. These in turn affect the effectiveness of any vaccine in improving population health.

COVID-19 is a case in point, as a classic example of a major transboundary anxiety-inducing crisis that directly puts at risk two nearly universally valued goods (good health and life), and which is best tackled primarily through international cooperation and collective, rather than merely private, action. Like other large-scale emergencies and disasters, the catastrophic nature of the pandemic outbreak is likely to ‘draw scrutiny from a wide range of citizens, not just those normally interested in news and politics’ (Atkeson & Maestas, 2012: 2–3). The institutional building blocks used for designing international COVID-19 alliances are therefore likely to be politically highly consequential.

A global race to develop, mass produce and distribute a vaccine against the COVID-19 pandemic started around early 2020. Ethical debates have raged about the general criteria by which scarce vaccines should be distributed (Ezekiel et al., 2020; Persad et al., 2020). These debates tend to focus on moral arguments for prioritizing between citizens within the same country. They pay less attention to the political feasibility and societal acceptance of specific international vaccine alliance formats. So far, we know very little about which particular type of international vaccine alliance citizens prefer. In a vaccine alliance, the purchasing power of its members is pooled to gain access to a vaccine that individual members would have difficulty procuring independently or could only procure at a higher price. The international vaccine alliance considered here is thus different from global health partnerships working with donor governments to increase access to vaccines for developing countries, such as GAVI or COVAX.^1^ How do citizens’ reason regarding the specific building blocks of COVID-19 alliances and the vaccine they produce? Such design formats matter for political support. Citizens tend to adjust their behavior and cooperation to normative cues they pick up regarding such institutional designs (Gibson & Nelson, 2014; Tankard & Paluck, 2017; Yair et al., 2020). How citizens evaluate international cooperation efforts appears to be driven at least in part by how they evaluate the specific characteristics and outcomes of these cooperative efforts (Bernauer et al. 2020; Bernauer & Gampfer, 2013; Dellmuth & Tallberg, 2015). This article aims to shed light on how informational cues about the design principles of COVID-19 vaccine alliances affect German citizens’ support for such alliances.

## ‘When no solutions have been found, yet the worst seems to be over’: a cognitive moment in pandemic cycles

A key property of our research design is the particular stage of pandemic cognition at the time of the fieldwork. In mid-summer 2020, when our survey experiment was conducted, a triple set of cognitive features—‘deep ignorance, high attentiveness, and false safety’—characterized the pandemic situation in Germany. First, there was still a very large degree of ignorance about the pandemic, as the larger public at that time had no information about either the state of development of any COVID-19 vaccine, the identity of the key vaccine producers, the state of play in negotiations regarding international vaccine alliances, or their nature. While the public was aware that various companies were working on developing a COVID-19 vaccine and that governments had entered negotiations with these companies, the critical parameters of pandemic policy solutions (i.e., when, from whom, and to what extent the vaccine would be available and how effective it was going to be) were still unknown. Second, COVID-19 was still relatively new and therefore attracted widespread press coverage and popular attention. Appendix Fig. 1 illustrates the large gap between the popular focus on the solution and on the disease itself at the time of our survey. As Google Trends shows, searches for the term ‘COVID Disease’ were at their highest level since the pandemic outbreak in Germany, whereas searches for the term ‘COVID Vaccine’ were even lower than at the outbreak.

**Fig. 1:**
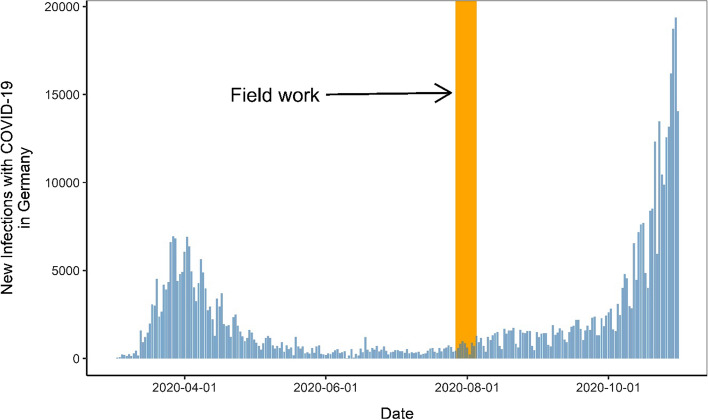
Number of new COVID-19 infections over time in Germany. Data are retrieved using the tidycovid19 R-package which downloads data from Johns Hopkins University on daily new COVID-19 infections for each country. Data display only number of new COVID-19 infections for Germany. Orange area displays the time period in which the survey experiment was conducted (end July/beginning of August 2020)

Third, despite this high attentive salience, there happened to be a very low number of new infections at that moment. As Fig. 1 shows, during our fieldwork, the number of new infections—arguably the critical variable deriving governments’ policy responses and media coverage of COVID-19 in the first year—was at its lowest in Germany since the first wave of the pandemic outbreak in March 2020. Even though no manageable policy solutions were known to have been reached, the pandemic was thus likely to have lost its initial ‘high emergency’ or ‘catastrophic’ anxiety-inducing factor (Atkeson & Maestas, 2012). Nor did the population realize that a much more devastating second wave was only two months away, starting in October and peaking around Christmas 2020.

In other words, our field work was conducted during a phase within the pandemic cycle when the very low number of new infections likely contributed to a false popular sense of safety or relief, or of ‘the worst already being over now.’ This false sense of safety may have been further driven by the specific location of the COVID-19 outbreaks that occurred during mid-summer 2020 in Germany, as these events were heavily locally concentrated at that time, notably in meat processing factories and the meatpacking industry (BBC, 2020).

## Multidimensional preferences regarding the institutional design of vaccine alliances

Citizens’ attitudes toward public policy generally vary greatly as a function of the specific features of the policy in question (Bansak et al., 2017; Bechtel & Scheve, 2013; Bechtel et al., 2017; Häusermann et al., 2019). However, on some, seemingly more ‘fundamental,’ policies, such as basic moral values or religious beliefs, there is much less evidence of such contingent preferences. Here, citizens often hold remarkably non-contingent views in response to variation in policy design. Regarding international COVID-19 vaccine alliances, citizens’ preferences are expected to be organized along four key building blocks that capture the key features of such alliances: (1) alliance composition (size; EU-centrism), (2) alliance distribution rules (joining cost; vaccine allocation), (3) vaccine nationalism (cost per German household; coverage in Germany), and (4) vaccine producer confidence (origin; type). Table 1 shows the four dimensions and value options used in the conjoint experiment. A second research question regards the degree of stability or non-contingency of citizens’ views on COVID-19 vaccine alliances as the latter’s design features vary. We ask whether multidimensional preferences for the design of an international vaccine alliance are influenced by respondents’ ideological self-placement or subjective perception of their personal risk of catching COVID-19.

**Table 1:** Policy dimensions and attributes of international vaccine alliances

Alliance composition (size; EU-centrism)	
Members of the alliance are Germany and	(1) 3 other states (2) 14 further states (3) 26 further states
The other members of the alliance are	(1) Exclusively EU states (2) EU states and other developed democracies (3) Predominantly non-EU states including non-democracies
Alliance distribution rules (joining cost; vaccine allocation)	
Distribution of costs within the alliance	(1) Rich states pay more than poor ones (2) Each state pays the same amount (3) Proportional according to population size
Distribution of the vaccine within the alliance	(1) According to population size (2) According to medical need (COVID19 cases). (3) according to financial participation
Vaccine nationalism (own-nation coverage; cost per national household)	
Vaccine doses for Germany in million units (population coverage in brackets)	(1) 12 (about 15% of the German population) (2) 33 (about 40% of the German population) (3) 58 (about 70% of the German population) (4) 82 (about 100% of the German population)
One-off costs per German household	(1) 33 Euro (2) 99 Euro (3) 166 Euro (4) 298 Euro
Vaccine producer confidence (origin; type)	
Vaccine manufacturer comes from	(1) China (2) USA (3) Germany (4) Switzerland (5) Great Britain
Vaccine is produced by	(1) A public university (2) A pharmaceutical company (3) A partnership between public university and pharmaceutical company

*Question* Which of the two vaccine alliances should Germany participate in? [Vaccine Alliance A vs. Vaccine Alliance B]

### Alliance composition

Which types of partner countries are likely to be preferred for building a vaccine alliance? Like other clubs aiming to solve collective action problems to provide valuable and costly goods, the number and provenance of vaccine alliance members are subject to strategic choice (Yi and Marathe 2015; Buchanan, 1965; Sandler, 1992). Selecting some alliance partner countries over others is a multidimensional choice, as potential partner countries vary with respect to economic size, culture, political system, and other attributes (Spilker et al., 2016). At least from the point of view of citizens in a large European country such as Germany, two attributes of alliance composition are likely to be critical: *size* as an indication of scale and scope, and *EU-centrism* as a proxy of cultural and political-geographic proximity and ‘psychic closeness’ (Spilker et al., 2016). In our study, the alliance composition question regards the number of other countries in addition to Germany and whether these countries are fully, predominantly, or only partly EU-based. The options for alliance size are small (three additional European countries), medium (14 further countries), and large (27 more countries). The maximum value of 27 is chosen to be consistent with the second attribute (‘EU-centrism’). Here, we distinguish between alliances that include only EU states (maximum 27), predominantly EU states, or also non-EU and non-democratic states.

### Alliance distribution rules

COVAX and related initiatives aiming at more equitable access to COVID-19 vaccines call for international solidarity, typically in the form of donations from vaccine-rich to vaccine-poor countries. A prior goal, however, is the production and procurement of a vaccine in the first place. A second building block of vaccine alliances thus concerns the distribution of costs and benefits. What are citizens’ procedural preferences regarding the way in which particular vaccine alliance formats allocate the costs of vaccine production and the benefits of the vaccine end product? Here, we distinguish two distributional/allocation conflicts: *joining cost* (the distribution of the financial burden of joining the alliance to produce the vaccine in the first place) and *vaccine allocation* of the subsequent end product between the alliance members. Allocation according to medical need (COVID-19 cases) reflects a need-based justice principle (Konow, 2003), whereas allocation according to financial participation reflects a quid pro quo rule. A joining cost rule according to population size reflects social justice as proportionality (Cappelen & Tungodden, 2017); one by which richer states pay more than poorer ones reflects progressive international solidarity; and one by which each state pays the same amount reflects a naïve version of equal treatment.

### Vaccine nationalism

Vaccine nationalism captures a form of self-interested outcome-oriented preferences (Fidler, 2020). On the one hand, vaccine nationalism is an expression of the fundamental obligation of governments to protect their citizen’s health. On the other, it undermines the chances of a unified solution to a global health crisis. Vaccine nationalism consists of two attributes in our study: the number of vaccine doses for own-nation citizens (measured in million units and represented by German population coverage), and the one-off cost per German household. Coverage values for Germany are chosen to reflect a low coverage (about 15% of the German population), medium–low (40%), medium–high (70%), and complete coverage (100%). The costs per German household are chosen to reflect the price expected by experts (Reuters, 2020): a low (33 Euros), medium (99 Euros), high (166 Euros) and very high (298 Euros) price. When the survey was fielded, price pitches of the major pharmaceutical companies were between 50–60 US dollars per course. The numbers we have implemented are also scaled to represent 1%, 3%, 5% and 9% of the average net income per month (EUR 3314) in Germany in 2016.

### Vaccine producer confidence

The fourth building block, vaccine producer confidence, captures subjective concerns and heuristics and narratives used by citizens concerning the vaccine's quality, effectiveness, and health safety (Betsch et al., 2011; Haase et al., 2020), once it has been produced. In times of major crisis, popular opinion initially ‘rallies round the flag’ (Kritzinger et al., 2021). It then favors national solutions and often also leaders perceived to take fast and strong policy measures (Mueller, 1970). This is not unlike the home bias pattern observed in investment portfolio behavior (French & Poterba, 1991). Despite potential gains from international diversification, the home bias in national investment portfolios tends to increase stronger during a crisis (Gelos & Wei, 2005). The dimension vaccine producer confidence has two attributes in our study: the country of origin of the vaccine manufacturer and the type of ownership situation of the manufacturer. The values for the countries of origin reflect the race for a vaccine in summer-autumn 2020: China, USA, Germany, Switzerland, and Great Britain. While all these countries host vaccine developers with the potential to discover vaccines for COVID-19, citizens’ attitudes, trust, and prejudices toward safety standards adopted by different producer countries in the course of developing, mass producing, and distributing a vaccine may vary. The second attribute differentiates whether the vaccine is developed by a public university, a private pharmaceutical company, or a partnership between a public university and a pharmaceutical company.

## Design and sample

We analyze public preferences for the four alliance design dimensions by using a paired conjoint experiment (Hainmueller et al., 2014; Leeper et al., 2020). Respondents are confronted with two hypothetical international vaccine alliances and then have to state which of the two they would prefer. Both vaccine alliance profiles vary randomly with regard to the set of attributes described above (Table 1). All attributes of the vaccine alliance profiles within subjects, as well as the order of presentation of attributes between subjects, are varied simultaneously, allowing us to analyze the relative importance of different attributes. Online Appendix Fig. 2 shows an image of the conjoint experimental instructions. Each subject receives three conjoint tables. In addition to the conjoint experiment, the survey also asked respondents how much they feel personally threatened by COVID-19.

**Fig. 2:**
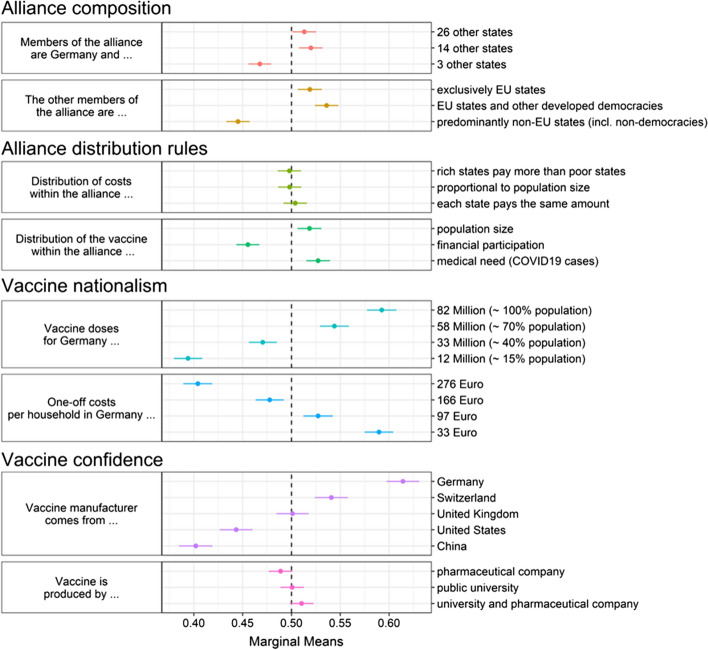
Effect of alliance attributes on public support for joining an international vaccine alliance

We surveyed 2285 eligible voters in Germany. The international survey firm Respondi recruited respondents from the population of eligible voters, to whom the survey was then administered online. We used quotas for age, gender, and region to avoid any lack of balance of the sample with regard to these covariates. Online Appendix Table 1 provides descriptive statistics. Informed consent was obtained from each participant at the beginning of the survey. The survey started on July 27 and ended on August 6, 2020, about four months after the first lockdown. During that period, the general policy measures to fight the pandemic in Germany were rather mild (e.g., social distance of at least 1.5 m, hygiene measures, mouth and nose protection when moving in closed rooms). As noted, the number of new COVID-19 infections was at a nationwide low since the outbreak of the pandemic (RKI 2020). Importantly, it was still practically impossible to predict who would first succeed in bringing an effective COVID-19 vaccine to market and when. Nor could the particular severity and duration of the next COVID-19 wave be foreseen, even though it was just around the corner in fall 2020.

## Results

Figure 2 displays the baseline results of the conjoint experiment. Following Leeper et al. (2020), we present Marginal Means (MMs) for each attribute in the conjoint experiment on the x-axis. MMs describe the probability of a profile being selected when it contains a certain level of an attribute. In forced-choice conjoint experiments, where respondents have to state a preference for one of two profiles, a MM of 0.5 serves as point of reference as it is the baseline probability that a profile is selected when a level has no effect on the selection probability. A MM above 0.5 indicates that respondents evaluate the attribute as favorable. In contrast to Average Marginal Component Effects (AMCEs), MMs do not depend on a reference category. This is particularly helpful when comparing subgroups (Leeper et al., 2020).

Our baseline results can be summarized as follows. First, respondents display a high degree of EU-centrism. They are significantly more likely to choose a vaccine alliance composed either exclusively of EU states or of EU states and other developed democracies (no significant differences between these options). The opposite is true for alliances with predominantly non-EU states, including non-democracies. Interestingly, the size of the alliance seems to matter less to respondents, although they do prefer medium and large alliances. Small alliances consisting of Germany and three further countries receive significantly less support than larger alliances with either 14 or 26 further member states.

Second, the normative dimensions capturing vaccine allocation and joining cost allocation seem to matter little or not at all. Whether the vaccine alliance allocates joining costs to member states according to population size, financial capacity, or equally, has no effect whatsoever on alliance support. However, alliances that distribute vaccine units according to prior financial participation are clearly less popular than those that allocate according to population size or medical need.

Third, and most strikingly, self-interest in the form of nation-based reasoning about vaccine alliances rules supreme. The two attributes capturing self-interested outcome-oriented preferences have the single most important effect on alliance support, in straightforward linear ways. Support increases both with lower costs per German household and with a larger coverage for Germany. This indicates that citizens may not consider vaccine alliances in ethical or fairness terms, but rather in vaccine nationalism terms, as a matter of national survival.

Fourth, while the specific vaccine producer type has hardly any impact on alliance support, the country of origin of the manufacturer does play a role. Here, we can observe further indications of vaccine nationalism. Alliances that buy the vaccine from a Chinese or US company are less likely to be supported. Alliance support among Germans is higher for Germanic (German, Swiss) producers; it is highest for our respondent’s own nation (Germany).

**Fig. 3:**
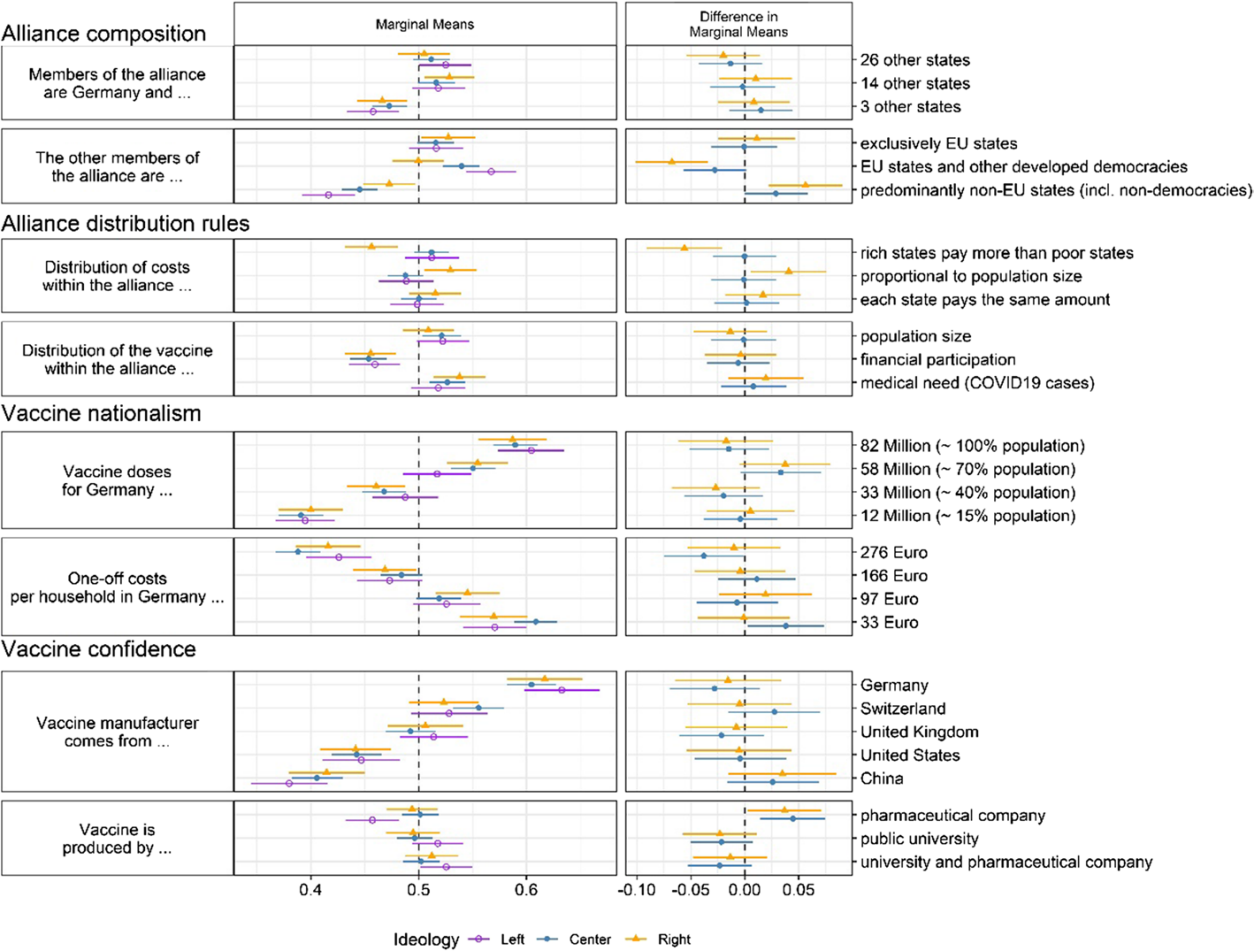
Effect of alliance attributes on public support for joining an international vaccine alliance by ideological self-placement

In a second step, we test for heterogeneous treatment effects. Specifically, we investigate whether multidimensional preferences for the design of an international vaccine alliance are influenced by (a) respondents’ subjective ideological left–right orientation (Baute & de Ruijter, 2021; Debus & Tosun, 2021) and (b) their perception of the personal threat of COVID-19 (Albertson & Gadarian, 2015; Gadarian et al., 2021). Figure 3 displays the Marginal Means conditional on the political ideology of a respondent (left, center, right). The three categories are based on recoding the continuous 11-point left–right self-placement variable. The right panel shows differences in Marginal Means, with left-wing respondents as reference category. There is remarkably little evidence of effect heterogeneity. The only exception can be found for the members of the alliance, where left–right ideology impacts respondents’ preferences regarding the inclusion of non-democracies. While all respondents are skeptical of this option (all Marginal Means < 0.5), right-wing respondents are less skeptical than left-wing respondents. Put differently, for left-wing respondents, the composition of the alliance seems to matter more.

**Fig. 4:**
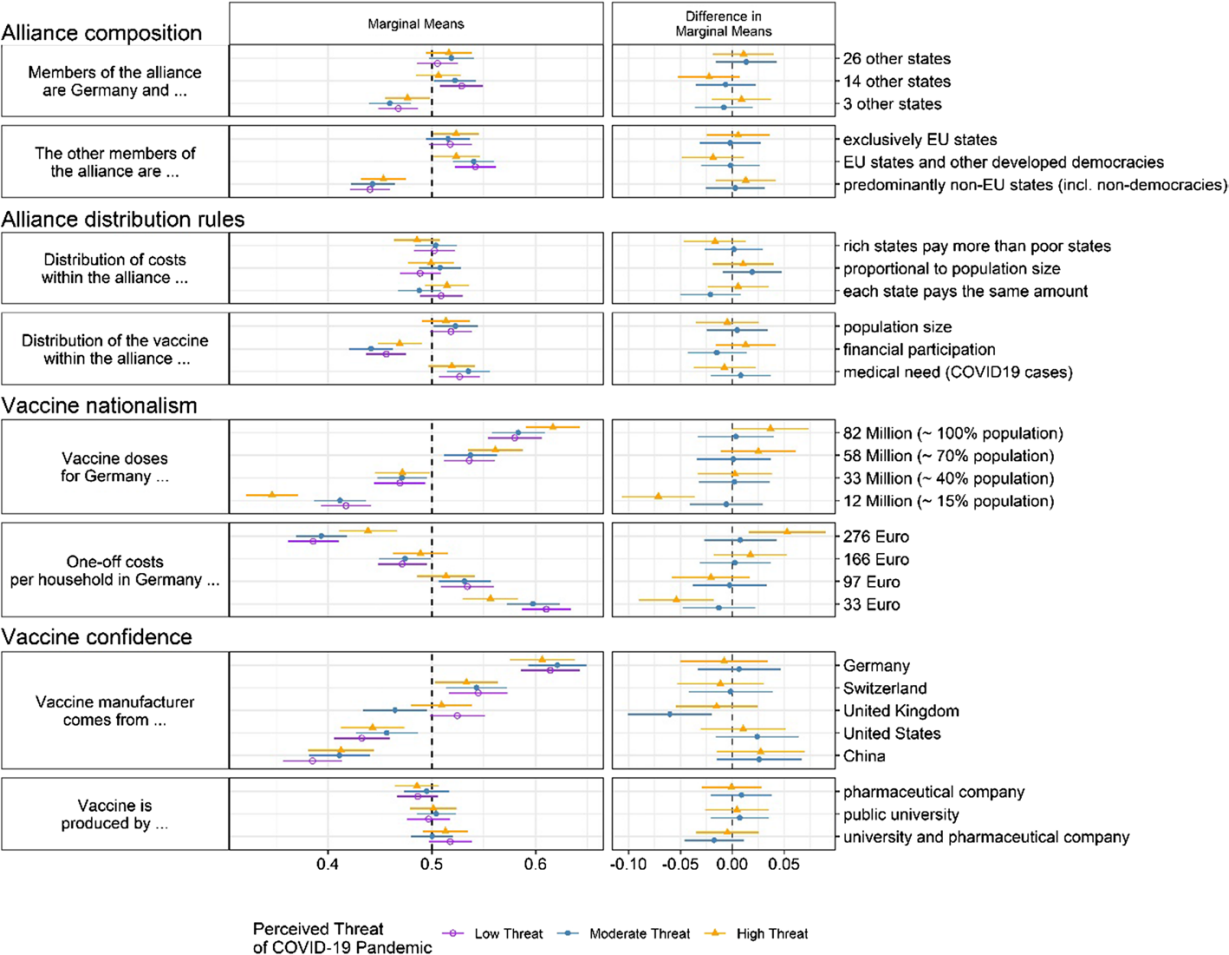
Effect of alliance attributes on public support for joining an international vaccine alliance by subjective threat

Figure 4 reports the effect of alliance attributes on public support for joining an international vaccine alliance by subjects’ perceived personal threat of the pandemic. For each attribute, three MMs are estimated, representing a low, moderate, and high subjective evaluation of the threat. Only in the case of vaccine nationalism do we see a moderating effect of perceived COVID threat. On the one hand, perceived threat amplifies the effect of national coverage: those who perceive the threat of COVID-19 as high are yet more likely to reject alliances providing lowest vaccine coverage levels for Germany (15 percent) and to support alliances providing full coverage. On the other hand, perceived threat diametrically tempers the baseline effect of cost per German household. That is, those with a high threat perception are distinctly less likely to be against vaccine alliances producing the costliest vaccine (276 euro per household) and, simultaneously, less likely to be in favor of alliances producing the cheapest vaccines (33 euro).

## Conclusions and implications: vaccine alliance design and pandemic politics

A better understanding of how popular support for international COVID-19 vaccine alliances depends on their specific design is important not just for the political feasibility of alternative policy choices but also for the subsequent effectiveness of vaccination campaigns – and therefore for population health. Such understanding is all the more important in light of the likely continuing mutations of the original COVID-19 virus to sometimes more lethal variants in the years to come. In an attempt to start the building of firmer micro-political foundations of COVID-19 cooperation support, this study has used a conjoint experimental design at a frequently recurring cognitive stage in pandemic cycles to explore Germans’ preferences regarding four key design building blocks: alliance composition, alliance distribution rules, vaccine nationalism, and vaccine producer confidence.

We have found that while a larger alliance size and dominant EU-state composition increase alliance support, national self-interest is even more important. Alliance support among Germans increases with both lower costs and larger coverage for national citizens. Vaccine nationalism, but also a form of Germanic-centrism, are also evident regarding vaccine producer origin: support goes down for Chinese and American producers, increases for Swiss producers and is highest for German producers. On the other hand, normative distribution rules regarding the allocation of alliance joining cost and final vaccine matter little or not at all. Somewhat surprisingly, citizens’ self-reported ideological orientation does not on the whole drive their vaccine alliance attitudes. Personal perceptions of the pandemic threat, lastly, also seem to play a lesser role, although the effect of national vaccine coverage for alliance support is amplified among those with a high treat perception. In sum, vaccine nationalism appears to reign supreme in the micro-politics of popular support for vaccine alliances at moments in the cycle when the worst already *seems* to be over, yet no policy solutions have been found. In addition to a science-driven, technocratic policy approach (Forster & Heinzel, 2021), a realist political outlook appears warranted at this stage, as there is scant foundational ground in popular preferences for constructing international cooperation based on rules of international solidarity.

The results reported here present a snapshot picture of citizens’ preferences for international vaccine alliances, yet they may offer wider lessons beyond the particulars of COVID-19 in Germany in mid-summer 2020. After all, the triple combination of ‘deep ignorance, high attentiveness, and false safety’ is a frequently recurring cognitive moment in the cycle of many major pandemics and other public health crises, and therefore a vital phase in disaster management. The lessons to be learnt from this snapshot are thus likely at once to be transferable within reason to other crises and to be specific to this cognitive moment in pandemic cycles. Policy-wise, the availability of an effective vaccine marks the entry of a new stage of the pandemic. Once a manageable solution is known to be available (in this case, the availability of an effective vaccine), very different strategic considerations kick in that interact with and overlay other motivations (e.g., behavior that might look like solidarity stems from national self-interest in preventing dangerous mutations in neighboring countries).

Our findings are mostly relevant for understanding popular attitudes toward international alliances in similar stages of future global health crises. Two main broader policy implications can be inferred, albeit with caution, from this study. First, the vaccine nationalism and the ‘vaccine home bias’ effects evident in our data may further contribute to nascent or re-emerging drives in many countries to renationalize key parts of the health sector and the pharmaceutical industry. Policymakers in many countries are likely to have learnt lessons from the early COVID-19 emergency in March 2020, when national public health resources were critically low and the dependency on foreign and private providers was high. During the Cold War, massive investments were made in West Germany and other West European countries to build and maintain significant extra operational capability to deal with potential large-scale public health emergencies due to military conflicts. With the end of the East–West conflict and the creeping marketization of the health sector in unified Germany, this institutional buffer capacity was significantly reduced, as became clear during the COVID-19 pandemic. It is likely that this experience, and the expectation of future emergencies caused by extreme weather, may contribute to the drive to rebuild such institutional buffer capacity. The transboundary nature of pandemics suggests that such policy initiatives should not focus exclusively on national-level policies for health- and disaster-preparedness. In addition, international crisis management institutions should also receive sufficient extra resources to maintain operational capability, even though these may present a case of organizational slack in non-crisis times. Future research, therefore, might further explore the policy options for government and citizens' preferences toward the design and the management of national and international health crisis and disaster-response institutions.

As regards distinct future stages in the pandemic cycle, any international cooperation on the procurement of future vaccines is not likely to emerge solely out of altruism or international solidarity. Ironically, the risk of dangerous virus variants that could destroy vaccine-rich countries' efforts to gain national herd immunity might increase these countries’ willingness to donate vaccines to poorer countries out of national self-interest rather than solidarity. Superficially, this study might be read to suggest that vaccine-nationalistic and Eurocentric vaccine alliances are the best strategy to prevent vaccination fatigue in the future. However, further research is still needed on whether the congruence between citizens’ institutional preferences and the design of vaccine alliances increases individuals’ willingness to get *vaccinated*. But our findings do suggest that policymakers would be well-advised to move carefully toward international vaccine alliances. The key to sustainable foundations for international cooperation, we speculate, might lie in sequencing and sheer good governance. If during the first emergency phase of pandemic cycles, governments’ primary goal may be to extinguish fires and protect their own populations, the effective pursuit of this goal may itself build legitimacy and increase popular support for more truly international cooperative solutions at later stages.

## Supplementary Information

Below is the link to the electronic supplementary material.

Supplementary file1 (DOCX 152 kb)

## Data Availability

Replication material (data and code) are available at the Harvard Dataverse: https://dataverse.harvard.edu/dataset.xhtml?persistentId=doi:10.7910/DVN/MJO2QZ.

## Footnotes

1 The distinction between vaccine buyers’ alliances and donor alliances is also important for answering how public support has been designed into the international COVID-19 vaccine alliances that have emerged at a later stage of the pandemic crisis cycle, say from early spring 2021 onward. As of summer 2021, the state of donor alliances still falls significantly short of expectations as COVID-19 vaccines are presumed to reach the poorest countries not before 2023 (Padma, 2021). The EU’s vaccine strategy most closely resembles a vaccine buyers alliance with elements of solidarity and burden-sharing among alliance members (Thibaud & Engeli, 2021). However, in contrast to the case studied in our conjoint experiment, the EU’s vaccine strategy involved the joint purchase of *several* vaccines from different suppliers.
