# Supplementary material for: Vaccine alliance building blocks: a conjoint experiment on popular support for international COVID-19 cooperation formats
Source: Policy Sci. 2021 Aug 11;54(3):493–506. doi: 10.1007/s11077-021-09435-1 (PMC8355869; doi:10.1007/s11077-021-09435-1)
Supplement: Supplementary file 1 — Supplementary file1 (DOCX 152 kb) [file 11077_2021_9435_MOESM1_ESM.docx]

**Online Appendix**

**Appendix Figure 1.** COVID-19 issue salience over time in Germany: Google Trends searches for 2 key terms


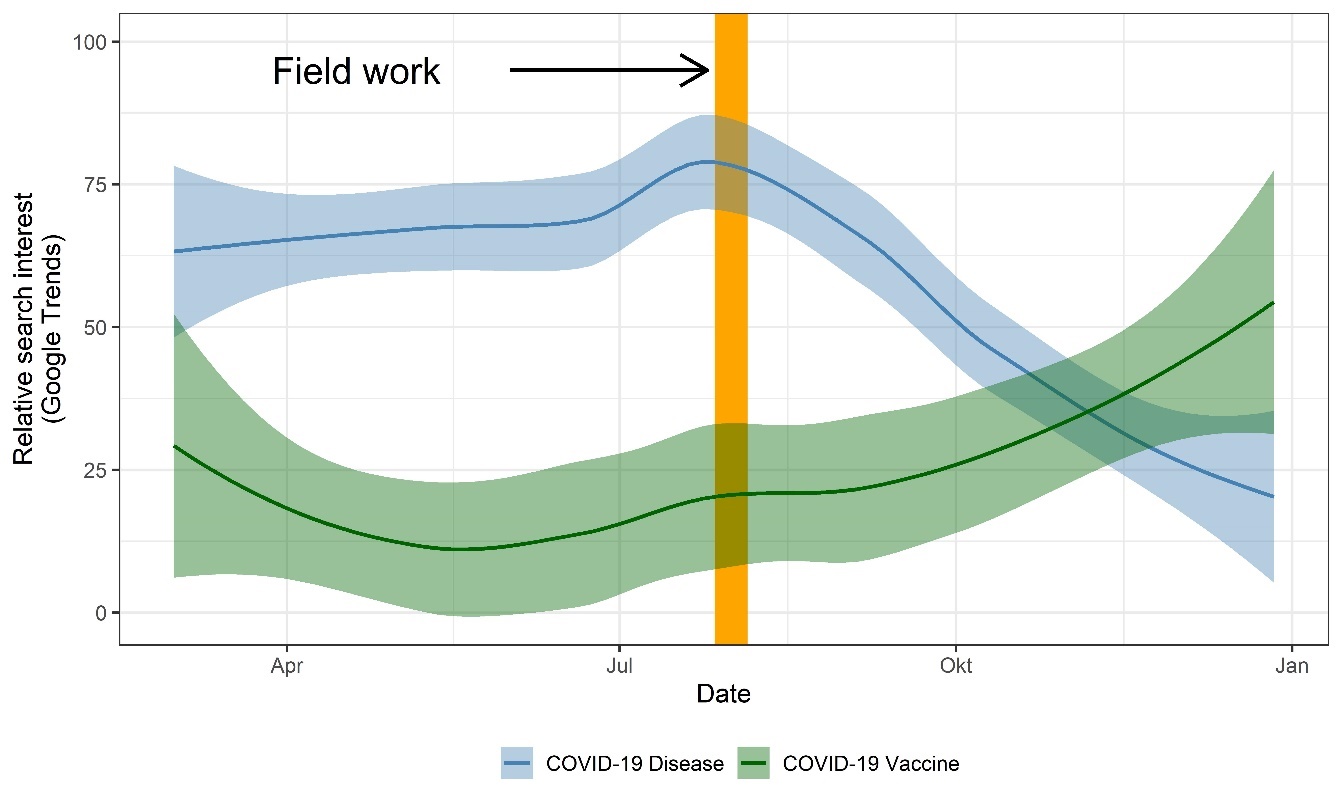


**Note:** Data displays google trends indicators from March 2020 to December 2020 for the search in Germany for the terms “COVID-19 Disease” and “COVID-19 Vaccine” (in German, “COVID-19 Erkrankung” and “COVID-19 Impfstoff”). Google Trends values have a range from 0 to 100, and indicate the search interest relative to the highest point in the chart for the selected region in the specified time period. The value 100 represents the highest popularity of this search term. The value 50 means that the term is half as popular and the value 0 means that there was not enough data for this term. Lines display loess-smoothers with 95% confidence intervals. Orange area displays time period in which the survey experiment was conducted (end July/beginning of August 2020).

**Appendix Figure 2.** Conjoint Introduction

A global race to develop a vaccine against COVID-19 is currently underway. In order to secure early access to a future vaccine against COVID-19, countries are forming so-called vaccine alliances. The countries of a vaccine alliance conclude a contract with a vaccine manufacturer and receive an exclusive quantity of vaccine doses at a predetermined price. There are different views on how such a Vaccine Alliance should be structured. In the following, we describe two possible vaccine alliances based on a number of characteristics and compare them in a table. Please take your time when comparing the two vaccine alliances. Then tell us which of the two vaccine alliances you think Germany should participate in. Here you can see how the table is structured.

| Feature | **Vaccine Alliance A** | **Vaccine Alliance B** |
| --- | --- | --- |
| Members of the alliance are Germany and | Shows the number of other states that are participating in the alliance. | |
| The other members are | Describes the type of states that participate in the alliance. | |
| Distribution of the vaccine within the alliance will be pro-rata | Shows how the vaccine doses are distributed among the members of the alliance. | |
| Distribution of costs within the alliance | Shows how the cost of the vaccine is distributed among the members of the alliance. | |
| Vaccine doses for Germany in million units (population coverage in brackets) | Shows how many doses of the vaccine Germany receives and what percentage of the German population can be protected. | |
| One-off costs per household in Germany | Shows what costs are incurred by German households. | |
| Vaccine manufacturer comes from | Shows from which country the vaccine manufacturer comes. | |
| Vaccine is produced by | Shows who developed and produces the vaccine. | |
| **In which vaccine alliance should Germany participate?** | O | O |

In total, we will present three of these tables, i.e. you will choose one of the two vaccine alliances three times. While the characteristics within the table change, the characteristics by which the vaccine alliances are described remain unchanged.

**Appendix Table 1.** Descriptive Statistics

| **Variable** | **Average** | **Median** | **Min** | **Max** |
| --- | --- | --- | --- | --- |
| Female | 0.50 | 0 | 0 | 1 |
| High Education | 0.36 | 0 | 0 | 1 |
| Age | 44.81 | 45 | 18 | 89 |
| Threat of COVID-19 | 2.88 | 3 | 1 | 5 |
| Left-Right | 5.70 | 6 | 1 | 11 |
| East Germany | 0.16 | 0 | 0 | 1 |
